# Linear scleroderma “en coup de sabre” with extensive brain involvement—Clinicopathologic correlations and response to anti-Interleukin-6 therapy

**DOI:** 10.1186/s13023-019-1015-7

**Published:** 2019-05-16

**Authors:** Cynthia M. Magro, Pierre Halteh, Luke C. Olson, Ilya Kister, Lee Shapiro

**Affiliations:** 1 Weill Cornell Medicine, Department of Pathology and Laboratory Medicine, 525 East 68th Street, New York, NY 10065 USA; 2 Weill Cornell Medicine, Department of Dermatology, 1305 York Avenue, New York, NY 10021 USA; 3 Department of Neurology, New York University Langone Medical Center, 240 East 38th Street, New York, NY 10016 USA; 4 Community Care Rheumatology, 1 West Avenue, Saratoga Springs, NY 12866 USA

**Keywords:** Linear scleroderma, “en coupe de sabre,” vasculitis, Autoimmune endotheliopathy

## Abstract

Linear scleroderma “en coup de sabre” (LSES) variant is a cephalic subtype of localized scleroderma that can be associated with extracutaneous stigmata, such as epilepsy, dementia syndromes, as well as focal central nervous system neurologic deficits. While the pathophysiology of cutaneous linear scleroderma includes endothelial cell injury and up regulation of pro-fibrogenic pathways, the basis of LSES-associated neurologic complications is largely unknown. We report a patient with a history of LSES who developed intractable epilepsy and cognitive decline. Magnetic resonance imaging (MRI) of the brain exhibited numerous persistently enhancing brain lesions. Due to progressive neurologic deterioration over a period of 7 years, despite interventional therapy, a brain biopsy was performed. Neuropathologic analysis exhibited acute and chronic cortical ischemia associated with a small vessel lymphocytic vasculitis. Direct immunofluorescent studies showed C5b-9 and IgG deposition on endothelium while indirect immunofluorescent studies demonstrated reactivity of the patient’s serum with the microvasculature of the patient’s own brain tissue and generic human umbilical vein endothelial cells indicative of anti-endothelial cell antibodies. Therapy focusing on damaged endothelium was implemented. The interleukin-6 (IL-6) receptor inhibitor tocilizumab was used and the patient improved dramatically, likely reflecting the drug’s effect on the replenishment of endothelial progenitor cells.

## Introduction

Localized scleroderma(LS), or morphea, is characterized by striking fibroplasia of the skin with frequent extension to the underlying subcutaneous tissues (i.e. morphea profundus) and bone(i.e. pansclerotic morphea) [1]. LS encompass 4 main categories: circumscribed morphea, generalized morphea, pansclerotic morphea and linear morphea, which is further subdivided into trunk/limb and head variants. The head variant termed “en coup de sabre” (LSES) is characterized by an indurated long streak resembling the deep wound of a sword. It appears as circumscribed linear or triangular induration in the fronto-parietal region, or less frequently, on nose, chin, cheek and neck [2].

LSES has been associated with a wide array of neurologic complications [3], neuropsychiatric syndromes, trigeminal neuralgia [4], hemiplegic migraines [5], and Rasmussen Encephalitis [6]. The clinical course may be progressive or self-limited. In most LS patients with neurologic symptoms, magnetic resonance imaging(MRI) of the brain shows one or more T2 hyper-intensities located in subcortical white matter, corpus callosum, deep gray nuclei and brainstem [3]. New brain lesions are observed in approximately half of the patients during follow up. MRI or cerebral angiogram may show features suggestive of vasculitis [3]. Brain pathological analysis of such cases is limited to a few anecdotal case reports with most descriptions reporting nonspecific inflammatory changes [3, 7]. While there is evidence for immune-based endothelial cell injury and up-regulation of pro-fibrogenic pathways in the pathogenesis of skin lesions of LS, the pathophysiologic basis of the central nervous system complications has not been elucidated [8, 9].

We present a 29-year female with LSES, intractable epilepsy, progressive cognitive decline and numerous enhancing brain lesions on MRI. This patient’s early course was reported by Kister et al. [3]. Herein, we present an additional 10 years of clinical follow up, including a period of progressive cognitive decline despite multiple immunosuppressive agents leading to a brain biopsy in 2014. We also describe the decision to use interleukin-6 (IL-6) receptor inhibitor tocilizumab in this patient, which resulted in a dramatic clinical improvement.

## Materials and methods

The patient underwent a brain biopsy whereby tissue was placed in formalin for routine histology and in physiologic fixative for immunofluorescent testing. Immunohistochemical assessment included myxovirus protein A (MXA), C3d, C4d, and C5b-9, while the direct immunofluorescent panel comprised IgG, IGA, IgM, C5b-9, C3d, C4d, C3 and C1q. The methodologies have been previously described [10].

Serum samples were evaluated for anti endothelial cell antibodies (AECA) by indirect immunofluorescent assay using permeabilized fixed endothelial cells derived from human umbilical cells incubated with a fluoresceinated human anti-IgG. AECA were also assessed via a Western blot technique using cutaneous endothelial cell lysates. Furthermore, the patient’s serum was directly incubated with the patient’s frozen brain tissue in the presence of fluoresceinated human anti-IgG to assess for the presence of circulating antibodies that could be directly binding to the patient’s brain tissue.

## Case report

**Fig. 1:**
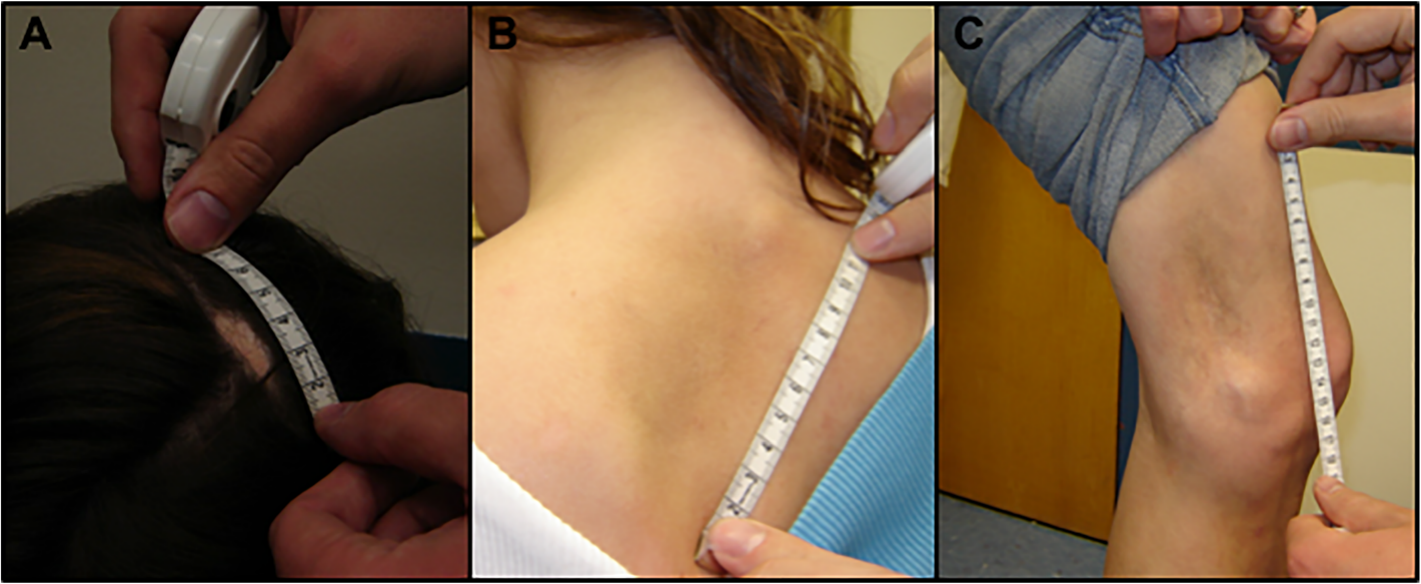
Clinical Images. **a** There is a striking linear area of alopecia and induration involving the vertex of the scalp defining a classic presentation of en coup de sabre. Additional areas of induration are present over the left superior back (**b**) and over the left distal medial left thigh (**c**)

**Fig. 2:**
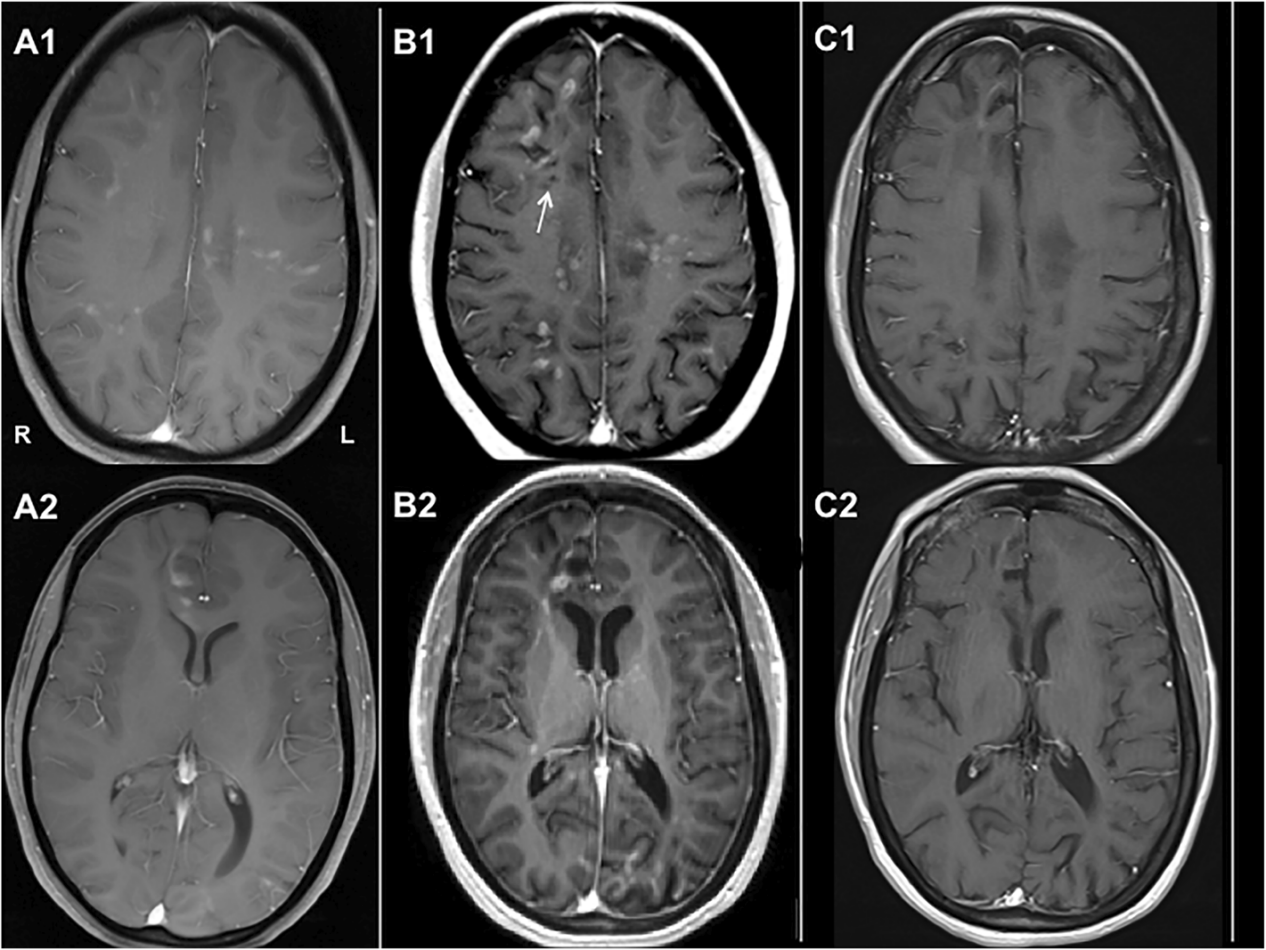
Neuroradiological Images. T1-weighted, Gadolinium enhanced images with the following findings: (**a1**, **a2**) Brain MRI at onset of neurologic symptoms shows patchy diffuse enhancement throughout both cerebral hemispheres and corpus callosum that involves cortex, juxto- and sub-cortical white matter. (**b1**, **b2**) Follow up brain MRI at the time of brain biopsy (6.5 years after onset) shows steady progression of disease with new areas of enhancement in frontal and occipital lobes as well as global cerebral volume loss (enlargement of ventricles, thinning of corpus callosum). Appearance of cavitary changes (T1 hypointensities, white arrow) suggests a more severe degree of brain injury. (**c1**, **c2**) MRI of the brain done after 7 months of tocilizumab therapy shows a remarkable decrease in the extent and number of previously enhancing lesions and no new enhancing lesions; widening of cortical sulci and ventricles is evident on follow up MRI

The patient’s pre-biopsy clinical history has been detailed previously [3]. In brief, her medical history was significant for herpes esophagitis at age 2, self-limited localized scleroderma- LSES, pansclerotic morphea, and superficial circumscribed morphea variants - at age 4, as well as migraines with aura and probable Raynaud’s disease in adolescence(Fig. 1). She was otherwise healthy, and at the time of onset of her neurologic symptoms, was on Dean’s List at her college. At age 22, she had first developed generalized tonic-clonic seizures and worsening migraines with aura. MRI of the brain at the time revealed a soft tissue and bone defect in the left parietal bone underlying the skin lesion, and over 25 enhancing lesions in bilateral juxtacortical, subcortical, and periventricular white matter and in the body of the corpus callosum (Fig. 2, **a1-a2**). Extensive serologic work up showed mild elevation in antinuclear antibody (ANA) (1:160, speckled). Anti-topoisomerase-1 (Anti-Scl-70-1) antibody, myositis specific- and myositis overlap- antibodies (anti-Jo-1, PL-7, PL-12, EJ, and OJ) were not immunoreactive.

The patient was diagnosed with central nervous system (CNS) inflammatory disease associated with LSES. Over the course of the next several years, her condition declined considerably despite treatment with repeated courses of IV high-dose methylprednisolone and IV immunoglobulin, plasmapheresis, a 6-month course of IV cyclophosphamide, a single dose of rituximab complicated by allergic reaction, oral methotrexate and azathioprine. The patient’s epilepsy became intractable even on multiple anti-epileptics, and cognitive deficits had progressed to the point where she could no longer live independently. Neuro-psychologic evaluation prior to brain biopsy showed attention, processing speed, expressive language, visuospatial functioning, and memory to be significantly below expectations, with relative sparing of receptive language and problem solving function (tests administered: Wechsler Abbreviated Scale of Intelligence (WASI-II); Wechsler Test of Premorbid Functioning (TOPF); Wechsler Adult Intelligence Scale-IV (WAIS-IV), Digit Span subtest; Repeatable Battery for the Assessment of Neuropsychological Status (RBANS); Verbal Controlled Oral Word Association Test (COWAT); Multilingual Aphasia Examination, select subtests; Trail Making Test (TMT); Wisconsin Card Sorting Test-64 item (WCST); Beck Depression Inventory (BDI-II); Beck Anxiety Inventory (BAI); Minnesota Multiphasic Personality Inventory (MMPI-2-RF)). She also experienced significant depression and anxiety, and was prone to anger outbursts. In parallel with clinical decline, serial MRI of the brain showed an increasing number of enhancing lesions with persistence of older lesions despite immunosuppression over the course of eight-year period (Fig. 2, **b1-b2**). Given the inexorably progressive course, a decision was made to pursue stereotactic brain biopsy to better understand the underlying pathology and attempt to formulate a more targeted approach to treatment.

## Results

### Routine histology

**Fig. 3:**
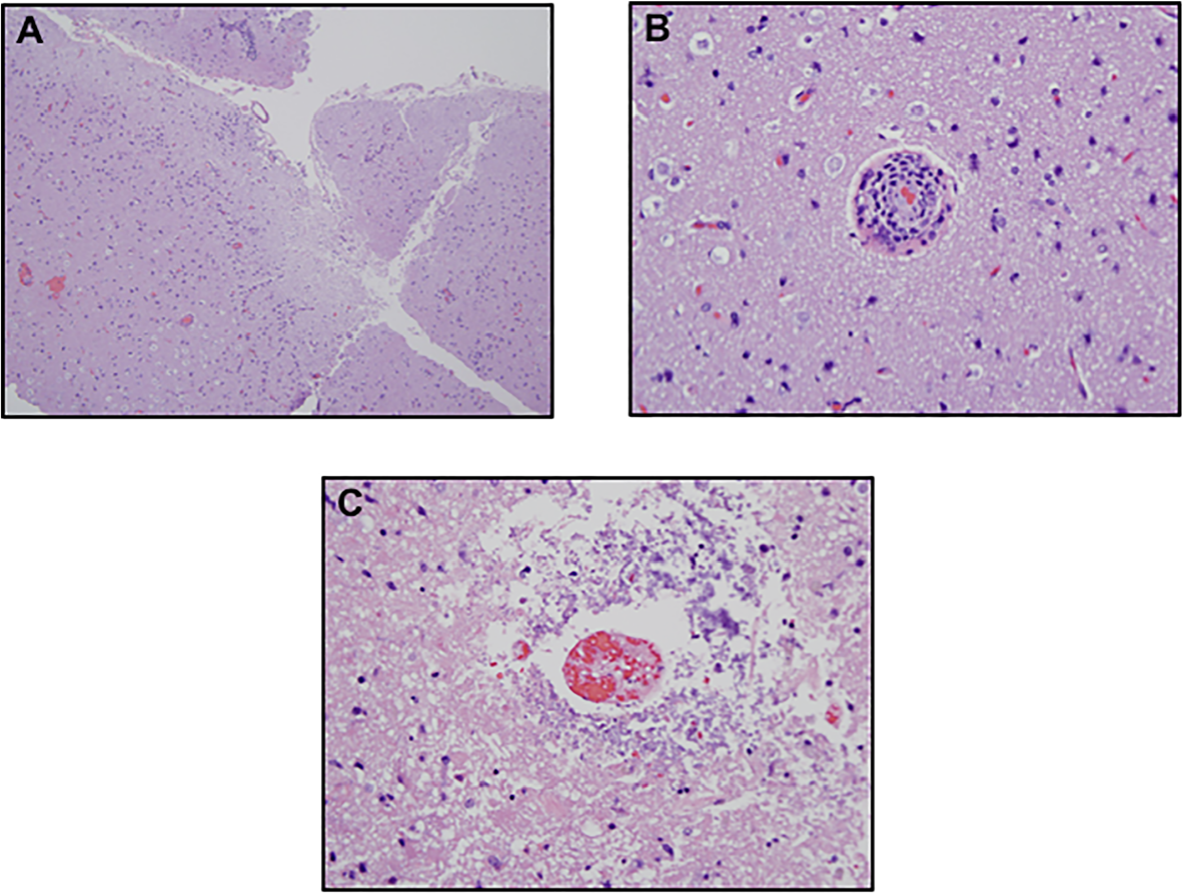
Neuropathological Findings with Hematoxylin and Eosin Staining. **a** The biopsy of the brain shows discrete areas of cortical necrosis. **b** Higher power magnification demonstrates a lymphocytic vasculopathy **c** pauci-inflammatory vascular thrombosis attributable to immune based endothelial cell injury. (**a**. Hematoxylin-eosin (H&E), 2x; **b**. H&E, 40x; **c**. H&E, 40x)

On microscopic examination, hematoxylin and eosin (H&E) stained material of the brain cortex demonstrated areas of cortical necrosis(Fig. 3a) associated with lymphocytes surrounding and permeating capillaries and venules of the meninges and brain cortex(Fig. 3b). In some vessels, there was vascular thrombosis without a significant angiocentric lymphocytic infiltrate(Fig. 3c). There was prominent endothelial cell swelling as well as a number of the vessels exhibited basement membrane zone reduplication reflective of antecedent episodes of vascular injury (not illustrated). Aside from the angiocentric inflammatory foci, there were also lymphocytes present within the brain parenchyma, infiltrating around glial cells, associated with cortical necrosis.

### Immunohistochemistry

**Fig. 4:**
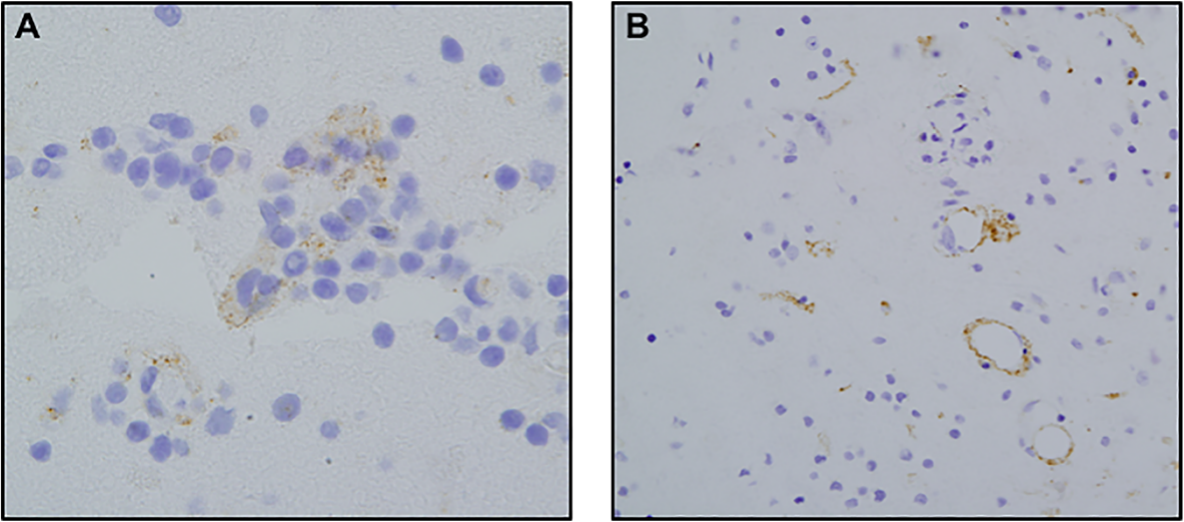
Neuropathological Immunohistochemical Staining on Paraffin Embedded Formalin Fixed Tissue: **a** A MXA preparation highlights endothelium (× 100). **b** There is staining of vessels for C4d, a stable component of classic complement activation, consistent with a Gell and Comb’s type II immune reaction targeting endothelium (× 100)

The MxA, a surrogate marker for the type I interferon microenvironment, demonstrated positive staining of endothelium(Fig. 4a). The C4d stable complement studies showed significant immunoreactivity within the microvasculature indicative of classic complement activation(Fig. 4b). The lymphocytic infiltrate was categorized immunohistochemically. The dominant cell populace was of T cell lineage as revealed by the degree of staining for CD3 with minimal staining for CD20. There was a relative increase in CD8-positive T cells, with an overall CD4 to CD8 ratio of approximately 1:2 due to the relative abundance of CD8 T cells compared to those of the CD4 subset.

### Direct immunofluorescent studies

**Fig. 5:**
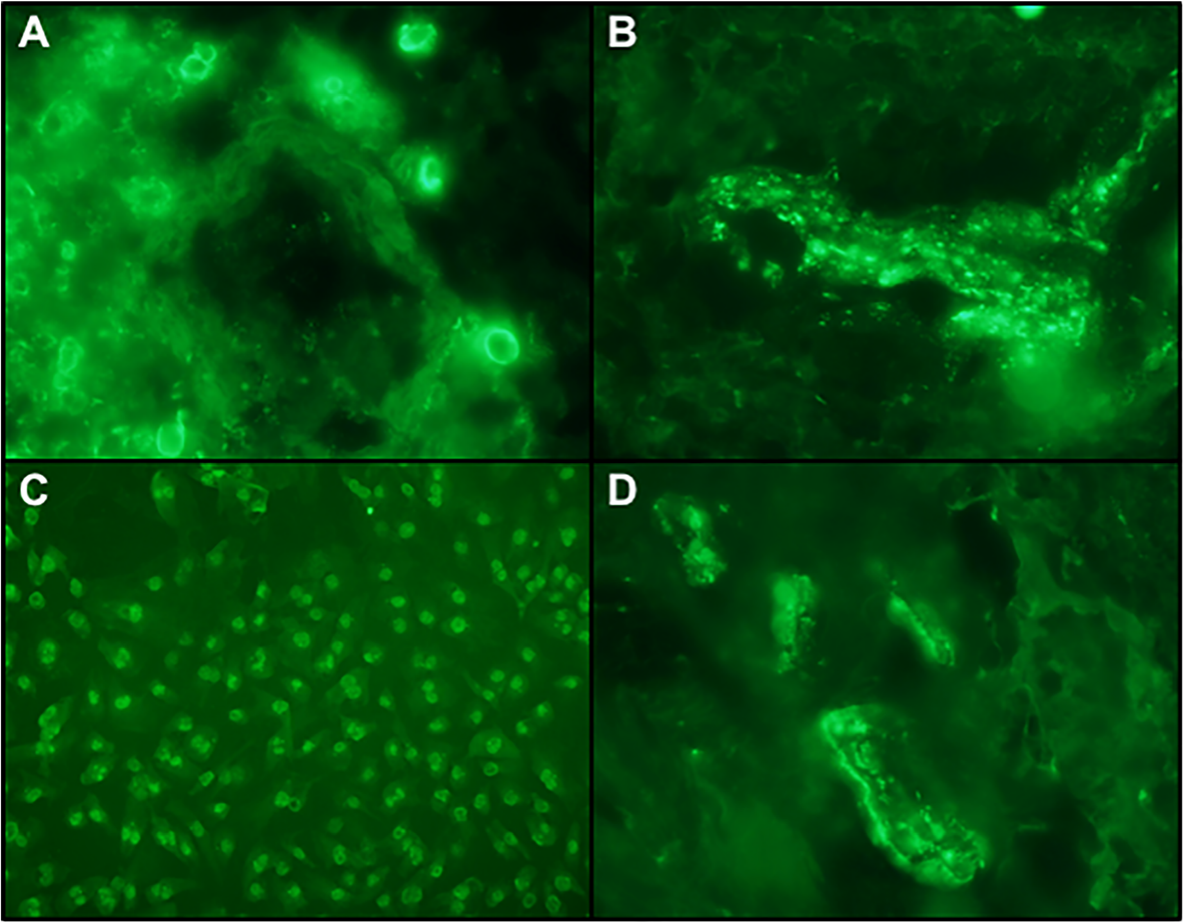
Neuropathological Findings: Direct and Indirect Immunofluorescent Studies. **a** There is deposition of IgG within the blood vessels showing direct endothelial cell localization indicative of antibodies of IgG isotype targeting the endothelium (× 100) (+ 3/3 staining intensity) **b** IF Studies showing striking reactivity for C5b-9 in blood vessels; C5b-9 is the effector mechanism of autoimmune endothelial cell injury syndromes including dermatomyositis, Kohlmeier-Degos disease in the setting of collagen vascular disease and Susacs syndrome (× 100)(+ 3/3 staining intensity). **c** The indirect immunofluorescent studies to assess for antiendothelial cell antibodies showed striking nuclear reactivity of generic endothelial cells with the patient’s serum. The pattern of reactivity suggests reactivity of generic endothelial cells with a para anti-centromere pattern using conventional Hep-2 substrates, (× 100) (+ 3/3 staining intensity). **d** Patient serum was incubated with her brain and showed the same pattern of reactivity as by DIF. Background cells are positive, showing a distinct scleroderma-like pattern (× 100) (+ 3/3 staining intensity)

Direct immunofluorescence studies performed on frozen brain tissue showed a prominent granular and homogeneous deposition pattern for IgG(Fig. 5a), IgM, C3, C3d, C4d, C5b-9(Fig. 5b), and fibrinogen within vessel walls and decorating endothelium. Smaller vessels in the capillary and venular size range were affected(Fig. 5a and b).

### Anti-endothelial cell antibody assay

Incubation of the patient’s serum with generic cutaneous endothelial cells revealed striking granular nuclear staining within the endothelium, consistent with a positive antiendothelial cell antibody assay (Fig. 5c).

### Indirect immunofluorescent assay utilizing patient’s serum and patient’s brain biopsy material

Incubation of patient’s serum with her frozen brain tissue revealed a positive reaction with granular nuclear staining of endothelial cells(Fig. 5d).

### Case history subsequent to biopsdy

Upon review of brain biopsy, a decision was made to pursue treatment with anti-complement therapy (eculizumab), however this drug could not be obtained on compassionate use basis. Patient was started on IL-6 receptor inhibitor tocilizumab (Actemra, Genentech USA, Inc., South San Francisco, CA) monotherapy, with titration of the dose to 162 mg weekly subcutaneous injections. The choice of toculizumab was made in view of successful experience with this agent in systemic scleroderma, especially in early disease when endothelial dysfunction and acute inflammation play a significant role in disease pathogenesis. Within one year of starting tocilizumab, there was a noticeable improvement in cognitive and affective symptoms with decrease in seizure frequency despite lower doses of three anti-epileptic agents. There was also a remarkable resolution of many of the enhancing lesions on brain MRI (Fig. 2, **c1-c2**). After 18 months of therapy, the patient was able to start working part time as a pre-school teacher assistant.

## Discussion

We have presented a detailed neuropathologic analysis of brain lesions associated with LSES. Our patient exhibited evidence of autoimmune vasculitis typical for a Gell and Comb’s type II immune reaction targeting endothelium. The pattern of endothelial cell injury oftentimes accompanied by vascular thrombosis and a variable angiocentric lymphocytic infiltrate defines the prototypic morphology encountered in the microvascular syndromes attributable to an anti-endothelial cell antibody syndrome, such as dermatomyositis [11–13], systemic lupus erythematosus [14] and possibly Susac syndrome [15]. The histopathology of these syndrome is also similar to that seen in this case being a combination of lymphocytic vasculitis, pauci-inflammatory thrombosis and chronic microvascular changes manifested by basement membrane zone reduplication and vascular ectasia. Further evidence of an autoimmune endotheliopathy syndrome targeting the brain vessels was revealed by immunohistochemical studies utilizing C3d and C4d on paraffine embedded tissue and by direct immunofluorescence showing deposits of complement including C3d, C4d, C5b-9 and IgG within vessels. In addition, the indirect immunofluorescent studies showed direct reactivity of the patient’s circulating IgG with the patient’s brain endothelium and generic endothelial cells, the latter showing a characteristic scleroderma centromere pattern.

It is probable that the critical effector of the endothelial cell injury in this case was the membranolytic attack complex(MAC) of complement, C5b-9 [16] which forms as a result of activation of either the classical or alternative pathways [17]. The C9 component deposits within the phospholipid bilayer through attachment to C5b-8 and forms tubular transport membrane channels in the surface membranes of target cells [18]. The resulting loss of membrane integrity causes cell injury and death.

The up-regulation of interferon alpha with localization to the endothelium in our patient is a finding encountered in Kohlmeier-Degos disease(malignant atrophic papulosis), dermatomyositis, systemic lupus erythematosus, and Aicardi-Goutieres syndrome, which is associated with a three prime repair exonuclease 1 (TREX1) mutation [16]. Type I interferon-rich microenvironment with vascular localization has not been reported in the setting of cutaneous LS, though there are reports of enhanced interferon alpha in severe scleroderma associated with microvascular complications in tissues [19, 20]. The up-regulation of MXA indicative of a strong type 1 interferon-rich microenvironment is seen in a subset of scleroderma patients with prominent microvascular disease [21].

The findings of a complement-mediated endothelial injury syndrome suggested that eculizumab, a humanized monoclonal antibody that prevents the cleavage of human complement component C5 into its pro-inflammatory components, would be a rational therapeutic choice for our patient [22, 23]. However, we were unable to obtain this drug, so we selected tocilizumab, a recombinant humanized monoclonal antibody, which acts as an IL-6 receptor inhibitor. IL-6 has been found to play a significant role in systemic scleroderma especially early in the disease when endothelial dysfunction and acute inflammation are postulated to be the main driving forces. IL-6 propagates chronic inflammation via its anti-apoptotic effect on neutrophils [24, 25] and T cells [26]. In patients with systemic scleroderma, elevated serum levels of IL-6 have been associated with the severity of skin fibrosis [27]. Fibroblasts isolated from lesions in scleroderma patients express higher levels of IL-6 [9]. Therefore, we hypothesized that IL-6 blockade with tocilizumab may abrogate some of sustained chronic inflammation in the brain of our patient.

IL-6 appears to play a role in propagating endothelial cell apoptosis in systemic scleroderma [28]. In the presence of neutrophils, serum from scleroderma patients significantly increases endothelial cell apoptosis and E-selectin expression, a leukocyte-endothelial adhesion molecule present on activated endothelial cells. These effects are partly IL-6-dependent, and depletion of IL-6 decreases levels of E-selectin, which abrogates endothelial apoptosis [8]. Endothelial activation and apoptosis lead to exposure of basement membrane extracellular matrix, specifically type IV collagen, with subsequent activation of the clotting pathway and vascular thrombosis. One study showed that toculizumab increased the population of endothelial progenitor cells (EPCs), a cell population responsible for vasculogenesis in adults [29]. Low levels of EPCs slow the recovery process of endothelial injury in rheumatoid arthritis and other thrombotic microangiopathic syndromes [30, 31], while higher levels of EPCs could promote repair after denudement of the microvasculature. We speculate that enhancing EPC pools may have promoted neurovascular recovery and repair of blood-brain barrier (as evidenced by resolution of contrast enhancement on MRI) in our patient.

## Conclusions

Our report illustrates the benefits of in-depth neuropathologic analysis to elucidate the pathophysiologic basis of injury in rare inflammatory disorders of CNS. To our knowledge, documentation of complement-mediated microvascular endothelial cell injury in brain lesions associated with LSES has not been reported previously. Our pathologic analysis suggests that treatment with anti-complement therapy with a drug such as eculizumab may be effective in such cases. It also helps explain why broad-spectrum immunosuppression (e.g. cyclophosphamide) that does not specifically target complement-mediated inflammation was not effective, while therapy with IL-6 receptor inhibitor yielded significant improvement. While it is important to refrain from over-extrapolation based on a single case, we hope that our work will stimulate further studies of neuro-inflammatory pathways in autoimmune diseases.

## Abbreviations

AECA: Anti endothelial cell antibodies
ANA: Antinuclear antibody
Anti-Scl-70-1: Antitopoisomerase-1 antibody
CNS: Central nervous system
EPC: Endothelial progenitor cells
H&E: Hematoxylin and eosin
IL-6: Interleukin-6
LSES: Linear scleroderma “en coup de sabre”
MAC: Membranolytic attack complex
MRI: Magnetic resonance imaging
MXA: Myxovirus-resistance protein A
TREX1: Three prime repair exonuclease 1
